# Medical Care Before and During the Winter Paralympic Games in Turin 2006, Vancouver 2010 and Sochi 2014

**DOI:** 10.1515/hukin-2015-0086

**Published:** 2015-01-12

**Authors:** Wojciech Gawroński, Joanna Sobiecka

**Affiliations:** 1 Department of Internal Medicine and Gerontology, Medical College, Jagiellonian University, Krakow; 2 Faculty of Motor Rehabilitation, University School of Physical Education, Krakow

**Keywords:** disabled sports, paralympic sports, quality of care, diseases, injuries

## Abstract

Medical care in disabled sports is crucial both as prophylaxis and as ongoing medical intervention. The aim of this paper was to present changes in the quality of medical care over the consecutive Paralympic Games (PG). The study encompassed 31 paralympians: Turin (11), Vancouver (12), and Sochi (8) competing in cross-country skiing, alpine skiing, biathlon and snowboarding. The first, questionnaire-based, part of the study was conducted in Poland before the PG. The athletes assessed the quality of care provided by physicians, physiologists, dieticians, and physiotherapists, as well as their cooperation with the massage therapist and the psychologist. The other part of the study concerned the athletes’ health before leaving for the PG, as well as their diseases and injuries during the PG. The quality of medical care was poor before the 2006 PG, but satisfactory before the subsequent PG. Only few athletes made use of psychological support, assessing it as poor before the 2006 PG and satisfactory before the 2010 and 2014 PG. The athletes’ health condition was good during all PG. The health status of cross-country skiers was confirmed by a medical fitness certificate before all PG, while that of alpine skiers only before the 2014 PG. There were no serious diseases; training injuries precluded two athletes from participation. The quality of medical care before the PG was poor, however, became satisfactory during the actual PG. The resulting ad hoc pattern deviates from the accepted standards in medical care in disabled sports.

## Introduction

The training load in contemporary competitive sports, including disabled sports, makes medical care indispensable. The regulatory changes leading to increased sporting competition caused a gradual shift from medical to functional classification and the creation of categories. As a result, victory has become largely related to physical fitness rather than the type of dysfunction or disability severity (Janke and Schule, 2006; Van de Vliet et al., 2011). The 2006 Paralympic Games (PG) in Turin were a breakthrough in disabled skiing where the number of events became limited by combining classifications. For the first time, medals were given in only three groups of athletes: standing visually impaired or blind athletes, and sitting or standing athletes with musculoskeletal impairment (Kroken and Lannem, 2003). This way, the smaller number of medals per event increased the competition, which in turn caused more intensive training leading to an increased risk of injury or overuse of the musculoskeletal system at each stage of the sporting career (DePauw and Gavron, 2005). Therefore, systematic medical check-ups should be an essential element of the training process in contemporary paralympic sport.

Medical check-ups are crucial (Best, 2004; Corrado et al., 2005; Crandell, 2007; Ljungqvist et al., 2009; Willick et al., 2011), but only thorough examinations and systematic follow-up can facilitate risk prevention, and may reveal a possible negative impact of the given sports discipline and training on the athlete’s body. In such a situation, a resolute and immediate medical intervention is necessary. The choice of an appropriate sports discipline or event must be very careful and personalised, depending on the disability type; a preliminary medical check-up must obligatorily precede training initiation (Gawroński, 1997). With appropriate medical care, sports training, as a form of physical activity of people with disabilities, is not only beneficial to the athletes and their health, but also to the community (Shephard, 1991). Winter sports, including skiing, may be seen as a continuation of rehabilitation as well as social, educational and professional integration with able-bodied people (Ferrara and Buchley, 1992; Sobiecka, 2004).

Unfortunately, disabled sport in Poland is no longer supervised by rehabilitation clinics and there is no medical care organisation system. Polish paralympians for many years have not been the subject of interest of sports medical institutions despite the increased disability-related risk (Sobiecka, 2005a). Only with the time, due to the influence of the “pathology” of contemporary competitive sports (intensification of training leading to overuse, the use of prohibited substances) on disabled sports, the need to provide medical care to athletes nominated to participate in the PG was acknowledged (Sobiecka, 2005b). With regard to able-bodied national representatives, guidelines drawn up by the physicians of the Central Sports Medical Outpatient Clinic in Warsaw were used. In 2003, after five years from the health care reform, the Health Ministry issued a Decree on the range and frequency of necessary medical examinations in children and adolescents under 21 applying for, or already in possession of, a licence for practicing amateur sport in a given discipline (The Official Journal of Laws of the Republic of Poland, August 13 2003; No 45: 391). As regulations regarding the obligation and range of medical examinations in athletes with disabilities were still lacking, the Decree first aimed to introduce such recommendations among winter paralympians. The Health Minister’s first Decree (The Official Journal of Laws of the Republic of Poland, January 8, 2007; No. 7: 56) on the range of medical care provided to the disabled athletes of the national team and the paralympic team, financed from the government budget, was only issued in 2007. At present, athletes with disabilities are included in the Health Minister’s Decree of 14th April 2011 on the range and ways of providing medical care to athletes qualified to the national team in Olympic and Paralympic sports (The Official Journal of Laws of the Republic of Poland, April 28, 2011; No. 8: 501).

The aim of the study was to present changes that took place in the standards of medical care during the preparations for the Winter Paralympic Games (PG) in Turin, Vancouver and Sochi, as well as during the actual PG.

## Material and Methods

### Participants

The study encompassed all the athletes nominated to participate in the PG in: Turin 2006 – 11 athletes (8 females, 3 males)*,* Vancouver 2010 – 12 athletes (9 males, 3 females) and Sochi 2014 – 8 athletes (8 males). The average age of respondents was in: Turin 32 ±8.7 years (22–51), Vancouver 30 ±10.3 years (17–55), Soczi 27 ±4.1 years (21–32). The athletes, with musculoskeletal and visual impairment, competed in: alpine skiing, cross-country skiing, biathlon and snowboard.

### Measures

The study made use of a two-part questionnaire by Kłodecka-Różalska adapted, with the author’s permission, for disabled sports. Part 1 of the survey contained 20 closed-ended questions in which the respondents were asked to assess the conditions during preparations for the PG on a scale from 1 to 5 (5 – excellent, 4 – good, 3 – average, 2 – low, 1 – very negative). Part 2 concerned the athletes’ socio-demographic and sports-related data (Sobiecka et al., 2012).

### Procedures

The study was composed of two parts and each comprised two stages. Part I concerned the athletes’ assessment of medical care during preparations for the PG. Part II focused on the medical events during the PG.

### Part I

*Stage 1* – the study was conducted in Poland during central training camps and consultations, approx. 4 weeks before leaving for the PG in: 2006, 2010 and 2014. All the study participants were given detailed instructions.

*Stage 2* – data concerning the medical aspect were extracted from the questionnaire. The respondents’ answers were categorised, and arithmetic means were calculated from the total of individual answers (scale 1 to 5) with regard to the conditions the athletes were provided with while preparing for the PG in 2006, 2010, and 2014. Next, based on the averaged assessment, three categories of conditions were identified: good (5.0-4.1), satisfactory (4.0-3.0), and poor (2.9–1.0).

### Part II

*Stage 1* – Preparticipation Health Evaluation (PHE). The athletes’ health condition was assessed min. 6 weeks before leaving for the PG and included a general medical check-up, orthopaedic examination, specialist consultations (laryngologist, ophthalmologist), resting ECG and laboratory tests (full blood exam, ESR, urinalysis). On successful completing of the evaluation, the athletes were issued a certificate stating the absence of medical contraindications to participating in the PG.

*Stage 2* – based on the documentation of the medical team physician during the PG in 2006, 2010 and 2014, all medical events (diseases and injuries) were analysed. Each medical event was presented separately in numbers and percentages.

All parts and stages of the study were performed following the code of ethics included in the International Ethical Guidelines on Biomedical Research involving Human Subjects published by the Council for International Organisations of Medical Sciences (CIOMS) together with the World Health Organisation (WHO), approved in 1982 and amended in 1993 and 2002 (International ethical guidelines for biomedical research involving human subjects. Geneva: Council for International Organizations of Medical Sciences, 2002 (access 15.06.2015)).

## Results

First, the conditions in which the Polish paralympians prepared for the three consecutive winter PG during central training camps and consultations were analysed. The analysis concerned: medical care, access to wellness services, cooperation with the massage therapist and the psychologist. The data show that not all athletes had access to medical care during preparations for the PG in 2006, 2010 and 2014 (Table 1).

The analysis of all means (=1.8; 3.0; 2.4) shows that the conditions were far from the athletes’ expectations. Before Turin, the conditions were poor according to alpine skiers (=1.8) and cross-country skiers, including biathlonists (=1.9). Differences in the assessment of cooperation with the physician were observed among athletes from the PG in Vancouver. Alpine skiers assessed their cooperation with the physician significantly better (=3.5) than cross-country skiers and biathlonists (=2.9). Before Sochi, negative opinions were expressed by alpine skiers ( =1.8) and cross-country skiers (=1.5). Only one snowboarder assessed the conditions as satisfactory ( =4.0).

Although access to wellness treatment after training and competitions was provided to alpine skiers in 2010 and to all paralympians in 2014, it was assessed as poor (= 2.5; =2.4, respectively) (Table 2).

All skiers from the Vancouver PG as well as one alpine skier form the Sochi PG had access to massage therapy (Table 3). The athletes’ assessment of their cooperation with the massage therapist changed from satisfactory in 2010 to poor in 2014.

Data concerning cooperation between athletes and the psychologist are presented in Table 4. Only alpine skiers had access to psychological care during preparations for the Turin and Vancouver PG. Before the Sochi PG, only one snowboarder had access to the psychologist. The athletes’ assessment of the quality of cooperation varied ranging from poor in 2006 (=2.8) to satisfactory in 2010 and 2014 (=3.6, =3.0, respectively).

Consultations with the dietician were not provided before any PG. Access to the physiotherapist was available only to alpine skiers in Sochi, and was considered satisfactory.

The analysis of the paralympians’ health condition based on the PPE did not reveal any absolute contraindications to participate in the PG.

Next, medical events that took place during the three consecutive PG were analysed (Table 5). Most interventions took place during the Turin PG (17), of which 11 were musculoskeletal injuries and six diseases. By contrast, there were no injuries during the Vancouver PG and only five diseases, mostly upper respiratory tract infections which did not preclude the athletes from participating in the competitions. In Sochi, there were only two musculoskeletal injuries needing medical intervention. Very importantly, over the three consecutive PG, there were only two musculoskeletal injuries that precluded the athletes from participating in the paralympic competition. Both took place during training, shortly before paralympic start, and their severity required helicopter transport.

The first injury took place in Turin 2006. An athlete with a lower-limb amputation suffered a lower leg fracture. The athlete was operated on and external immobilisation was applied. After six months from removing the fixation, the athlete resumed alpine skiing.

The second injury, a knee ligament injury, took place before the Sochi PG and was first treated conservatively, and on return to Poland, surgically.

The remaining injuries of the musculoskeletal system were chronic overuse lesions intensified during the PG.

## Discussion

Paralympic success is affected by the athlete’s preparation, intensive training during the preparatory period and high standards of medical care (Steadward et al., 2003). The source materials of the “START” Polish Disabled Sports Association show that disabled athletes participating in the winter PG before 1998, had no access to sport medical care at all (Sobiecka et al., 2011). In Nagano, for the first time paralympians were accompanied by a physician – a sports medicine specialist and a physiotherapist (Hady-Bartkowiak, 1998). This became a rule in the subsequent PG. Nevertheless, medical care was introduced only for the duration of the actual PG, and in Vancouver included two physiotherapists. By comparison, during the 1992 PG in Barcelona, Great Britain had a 12-member medical team composed of an orthopaedist, physiotherapists, nurses and a prosthetic specialist (Reynolds et al., 1994). Naturally, it is not about the size of the team, which is related to the number of athletes, but the actual composition of the medical team (Melion and Walsh, 1999).

Stress tests preceded by laboratory tests were first introduced before the PG in Nagano and only encompassed cross-country skiers. Between 2001 and 2010, cross-country skiers underwent relatively systematic (mostly once or twice/year) stress tests (Sobiecka et al., 2011). Each time, the tests were preceded by medical check-ups equivalent to the sports medicine check-ups performed in able-bodied athletes. Thus, cross-country skiers have had specialist medical care since 2002, unlike alpine skiers who first had such tests only in 2014. The range of examinations was first based on the recommendations of the Main Sports Medicine Centre, later on the Sport Minister’s Decree of 2007 r. (The Official Journal of Laws of the Republic of Poland, August 13 2003; No 45: 391), and now on the Health Minister’s Decree of 2011 (The Official Journal of Laws of the Republic of Poland, April 28, 2011; No 8: 501).

The importance of PPE has been acknowledged by the International Olympic Committee (IOC) in its Consensus Statement (Ljungqvist et al., 2009) and the International Paralympic Committee is planning a similar statement with regard to athletes with disability (Grimm, 2014). The role of examinations has been confirmed by epidemiological studies conducted during the consecutive Olympic Games and the PG in London (Schwelnus et al., 2013).

Observations of Gawroński et al. (2013) also confirm the impact of physical examinations on decreasing the number of diseases and musculoskeletal injuries during the London PG compared to the Beijing PG as a result of the rigorous execution of PPE before the summer PG in 2010. The results regarding winter sports athletes show a similar trend, i.e. the number of medical interventions over the consecutive PG decreases with systematically introduced medical check-ups. Indeed, comparing the 2006 and 2014 PG, diseases dropped from 6 to 2, whilst injuries from 11 to 2.

The athletes’ low awareness of the need to perform medical examinations is at odds with the results of the study, in which access to medical care was assessed negatively. On the one hand, the athletes are unwilling to undergo prophylactic health examinations, probably due to fear of exclusion from the PG (Sobiecka, 2005a; Reynolds et al., 1994). On the other hand, they often demand unreasonable supplementation or particular methods of physiotherapy. Therefore, continuing education of athletes in this regard is necessary.

Since 2011, medical examinations, treatment and rehabilitation of members of the national paralympic team have been offered free of charge, financed from the government budget (The Official Journal of Laws of the Republic of Poland, April 28, 2011; No. 8: 501). Nevertheless, the fact that the services are only provided at the Main Sports Medicine Centre in Warsaw may be a complication for athletes who live far from the capital city, especially winter sports athletes.

The presence of a medical team is very important in the training process, particularly with regard to athletes with disabilities. Unfortunately, during the studied period of winter paralympic preparations, there were no funds to create such a team. The available funds were allocated to the training camps only and barely covered ad hoc medical examinations and procedures. The range of wellness services depended on the training centre’s offer, while access to massage therapy depended on finding a volunteer massage therapist. In view of the above, there was no possibility to include a physiotherapist and a dietitian. Nonetheless, some alpine skiers had access to a volunteer sports psychologist, while cross-country skiers had a volunteer physician.

In the light of the above, the athletes’ negative opinions on the standards of medical care provided during preparations for the PG is hardly surprising. The negative feeling is intensified by the ad hoc character of the services provided and the absence of a well-organised medical team able to face the challenges of contemporary disabled sports. The analysis of the presented data demonstrates the organisational weakness of Polish disabled sports. Medical care provided only during the actual PG confirms the sports authorities’ lack of understanding of the situations and long-term perspectives.

## Conclusions

- The standard of medical care during preparations for the Paralympic Games is insufficient and far from the standards accepted in competitive sports;
- Medical care during the Paralympic Games has an ad hoc character and involves medical interventions on an as-needed basis;
- The introduction of systematic PPE is necessary to monitor the athletes’ health; strict execution of PPE tends to decrease the number of diseases and musculoskeletal system overuse.

## Recommendations

The recommendations that can be made based on the results of the survey and our own observations from the perspective of the three consecutive Winter PG are as follows:

- The organisation of a Medical Team coordinated by a physician, with the clinical and scientific support of a physiologist, a physiotherapist, a dietitian, a psychologist, an engineer and a biomechanics engineer;
- The national teams of each discipline should cooperate with a sports medicine specialist or, at minimum, with a physician (a specialist in orthopaedics and traumatology, medical rehabilitation, internal diseases) trained in sports medicine (min. introductory course for sports medicine specialisation) and in disabled sports;
- Selected teams should have continuous access to a physiotherapist familiar with sports medicine and experienced in disabled sports.

## Figures and Tables

**Table 1:** Analysis of access to medical care during paralympic preparations (athletes’ assessment)

Paralympians total			Assessment of conditions			
	
Sports discipline	n	%	Athletes using the services (n)	Mean assessment	Category of conditions*	Athletes without access (n)
**WINTER PARALYMPIC GAMES TURIN 2006**						
Alpine skiing	4	36.4	3	1.8	poor	1
Cross-country skiing and biathlon	7	63.6	3	1.9	poor	4
Total	11	100	6	1.8	poor	5
**WINTER PARALYMPIC GAMES VANCOUVER 2010**						
Alpine skiing	5	41.7	2	3.5	satisfactory	3
Cross-country skiing and biathlon	7	58.3	7	2.9	poor	0
Total	12	100	9	3.0	satisfactory	3
**WINTER PARALYMPIC GAMES SOCHI 2014**						
Alpine skiing	5	62.5	5	1.8	poor	0
Cross-country skiing and biathlon	2	25.0	2	1.5	poor	0
Snowboard	1	12.5	1	4.0	good	0
Total	8	100	8	2.4	poor	0

* Interpretation of mean assessment: 5.0 – 4.1 good; 4.0 – 3.0 satisfactory; 2.9 – 1.0 poor

**Table 2:** Analysis of access to wellness services after training and competitions during paralympic preparations (athletes’ assessment)

Paralympians total			Assessment of conditions			
	
Sports discipline	n	%	Athletes using the services (n)	Mean assessment	Category of conditions*	Athletes without access (n)
**WINTER PARALYMPIC GAMES TURIN 2006**						
Alpine skiing	4	36.4	0	0	n/a	4
Cross-country skiing and biathlon	7	63.6	0	0	n/a	7
Total	11	100	0	0	n/a	11
**WINTER PARALYMPIC GAMES VANCOUVER 2010**						
Alpine skiing	5	41.7	4	2.5	poor	1
Cross-country skiing and biathlon	7	58.3	0	0	n/a	7
Total	12	100	4	2.5	poor	8
**WINTER PARALYMPIC GAMES SOCHI 2014**						
Alpine skiing	5	62.5	5	2.8	poor	0
Cross-country skiing and biathlon	2	25.0	2	2.0	poor	0
Snowboard	1	12.5	1	1.0	poor	0
Total	8	100	8	2.4	poor	0

* Interpretation of mean assessment: 5.0 – 4.1 good; 4.0 – 3.0 satisfactory; 2.9 – 1.0 poor

**Table 3:** Analysis of access to massage therapy during paralympic preparations (athletes’ assessment)

Paralympians total			Assessment of conditions			
	
Sports discipline	n	%	Athletes using the services (n)	Mean assessment	Category of conditions*	Athletes without access (n)
**WINTER PARALYMPIC GAMES TURIN 2006**						
Alpine skiing	4	36.4	0	0	n/a	4
Cross-country skiing and biathlon	7	63.6	0	0	n/a	7
Total	11	100	0	0	n/a	11
**WINTER PARALYMPIC GAMES VANCOUVER 2010**						
Alpine skiing	5	41.7	2	3.0	satisfactory	3
Cross-country skiing and biathlon	7	58.3	7	3.0	satisfactory	0
Total	12	100	9	3.0	satisfactory	3
**WINTER PARALYMPIC GAMES SOCHI 2014**						
Alpine skiing	5	62.5	1	2.0	poor	4
Cross-country skiing and biathlon	2	25.0	0	0	n/a	2
Snowboard	1	12.5	0	0	n/a	1
Total	8	100	0	0	n/a	7

* Interpretation of mean assessment: 5.0 – 4.1 good; 4.0 – 3.0 satisfactory; 2.9 – 1.0 poor

**Table 4:** Analysis of access to psychological care during paralympic preparations (athletes’ assessment)

Paralympians total			Assessment of conditions			
Sports discipline	n	%	Athletes using the services (n)	Mean assessment	Category of conditions*	Athletes without access (n)
**WINTER PARALYMPIC GAMES TURIN 2006**						
Alpine skiing	4	36.4	4	2.8	poor	0
Cross-country skiing and biathlon	7	63.6	0	0	n/a	7
Total	11	100	4	2.8	poor	7
**WINTER PARALYMPIC GAMES VANCOUVER 2010**						
Alpine skiing	5	41.7	5	3.6	satisfactory	0
Cross-country skiing and biathlon	7	58.3	0	0	n/a	7
Total	12	100	5	3.6	satisfactory	7
**WINTER PARALYMPIC GAMES SOCHI 2014**						
Alpine skiing	5	62.5	0	0	n/a	5
Cross-country skiing and biathlon	2	25.0	0	0	n/a	2
Snowboard	1	12.5	1	3.0	satisfactory	0
Total	8	100	1	3.0	satisfactory	7

* Interpretation of mean assessment: 5.0 – 4.1 good; 4.0 – 3.0 satisfactory; 2.9 – 1.0 poor

**Table 5:** Medical interventions during the Winter Paralympic Games (based on the documentation of a medical team physician)

Medical interventions	Paralympians	

	n	%
**TURIN 2006**		
Injuries	11	64.7
Diseases	6	35.3
Total	17	100
**VANCOUVER 2010**		
Injuries	0	0
Diseases	5	100
Total	5	100
**SOCHI 2014**		
Injuries	2	100
Diseases	0	0
Total	2	100
